# Recessive variants in *WSB2* encoding a substrate receptor of E3 ubiquitin ligase underlie a neurodevelopmental syndrome

**DOI:** 10.1038/s41431-025-01863-4

**Published:** 2025-05-15

**Authors:** Shiyu Luo, Valérie Gailus-Durner, Bobbi McGivern, Qifei Li, Jessica Kottmeier, Mai-Lan Ho, Hagar Mor-Shaked, Orly Elpeleg, Erfan Aref-Eshghi, Amanda C. Brodeur, Klaus Schmitz-Abe, Casie A. Genetti, Jonathan Picker, Jiahai Shi, Reem Ibrahim Bux, Tawfeg Ben-Omran, Helmut Fuchs, Tamar Harel, Martin Hrabě de Angelis, German Mouse Clinic Consortium, Lillian Garrett, Oana Veronica Amarie, Nadine Spielmann, Adrián Sanz-Moreno, Patricia da Silva-Buttkus, Nathalia RV Dragano, Lore Becker, Sabine M. Hölter, Claudia Seisenberger, Susan Marschall, Juan Antonio Aguilar-Pimentel, Pankaj B. Agrawal

**Affiliations:** 1 Division of Neonatology, Department of Pediatrics, University of Miami Miller School of Medicine and Holtz Children’s Hospital, Jackson Health System, Miami, FL 33136 USA; 2 Division of Genetics and Genomics, Boston Children’s Hospital, Harvard Medical School, Boston, MA 02115 USA; 3 The Manton Center for Orphan Disease Research, Boston Children’s Hospital, Harvard Medical School, Boston, MA 02115 USA; 4 Institute of Experimental Genetics and German Mouse Clinic, Helmholtz Zentrum München, German Research Center for Environmental Health, Neuherberg, Germany; 5 GeneDx LLC, Gaithersburg, MD USA; 6 Division of Medical Genetics, Department of Pediatrics, University of Missouri School of Medicine, Columbia, MO 65212 USA; 7 Division of Neuroradiology, University of Missouri, Columbia, MO 65212 USA; 8 Department of Genetics, Hadassah Medical Center, Jerusalem, Israel; 9 Faculty of Medicine, Hebrew University of Jerusalem, Jerusalem, Israel; 10 Department of Child and Adolescent Psychiatry, Boston Children’s Hospital, Harvard Medical School, Boston, MA 02115 USA; 11 Department of Biochemistry, Yong Loo Lin School of Medicine, National University of Singapore, Singapore, Singapore; 12 Department of Adult and Pediatric Medical Genetics, Hamad Medical Corporation, Doha, Qatar; 13 Division of Genetic and Genomic Medicine, Sidra Medicine, Doha, Qatar; 14 Chair of Experimental Genetics, TUM School of Life Sciences, Technische Universität München, Freising, Germany; 15 German Center for Diabetes Research (DZD), Neuherberg, Germany; 16 Institute of Developmental Genetics, Helmholtz Zentrum München, German Research Center for Environmental Health, Neuherberg, Germany; 17 Technische Universität München, Freising-Weihenstephan, Munich, Germany

**Keywords:** Genetics research, Neurodevelopmental disorders, Disease genetics

## Abstract

WD40 and SOCS box protein-2 (WSB2), a member of the large family of suppressor of cytokine signaling (SOCS)-box proteins, has recently been identified as a substrate receptor of cullin 5 E3 ligase that plays an important role in proteomic regulation through substrate ubiquitination and proteasomal degradation. Here we report five patients from four unrelated families presenting with neurodevelopmental delay, dysmorphic features, brain structural abnormalities with or without growth restriction, hypotonia, and microcephaly, all of whom are homozygous for extremely rare and predicted loss-of-function (pLoF) or missense variants in *WSB2*, inherited from consanguineous parents. The *Wsb2*-mutant mice exhibited several neurological findings that included hyperactivity, altered exploration, and hyper alertness. They also weighed less, had a lower heart rate, and presented an abnormal retinal blood vessel morphology and vasculature pattern along with decreased total thickness of the retina. Our findings suggest that homozygous LoF *WSB2* variants cause a novel neurodevelopmental disorder in humans with similar neurologic and developmental findings seen in *Wsb2*-mutant mouse models.

## Introduction

Neurodevelopmental disorders (NDDs) represent a large group of disorders arising from alterations of tightly coordinated processes that regulate development and function of the brain, including intellectual disability (ID), autism spectrum disorders, and epilepsy [1]. With genomic advances, novel genes and related pathways are being identified at a rapid pace, but a significant gap continues to exist in our molecular understanding of NDDs.

Ubiquitination is one of the key regulatory mechanisms that control protein stability in a myriad of cellular processes, including neural development [2–4], and its disruption has been linked to many neurodevelopmental and neurodegenerative disorders [5–7]. The process of protein ubiquitination requires an enzymatic cascade that consists of a ubiquitin-activating enzyme (E1), ubiquitin-conjugating enzyme (E2) and an E3 ubiquitin ligase (E3). Although there are only 2 E1 and 30–50 E2 genes, the human genome encodes for >600 E3 ubiquitin ligases, which act post-translationally to regulate the activity and stability of the entire proteome [8]. The majority of E3s belong to the ‘really interesting new gene’ (RING)-type gene family. Among the RING-type ligases, Cullin-RING-type ligases (CRL) are the multi-subunit ligases whose major component is a specific cullin (CUL) molecule which binds to a RING-box protein (Rbx1 or Rbx2) and a substrate receptor (SR; via an adapter subunit in some cases) at its C- and N-terminus, respectively [9]. Since the original reports associating *UBE3A* with Angelman syndrome in 1997 [10], approximately fifty-five genes coding for either E3 ubiquitin ligases or CRL SRs have been identified as causative genes for fifty-eight different forms of NDDs [6, 11, 12]

The human *WSB2* (hg38; chr12:118,032,687-118,061,179) encodes for a CRL SR protein that contains seven WD-repeats (WD40) spanning most of the protein and a suppressor of cytokine signaling (SOCS)-box in the C-terminus. WSB2 belongs to a large family of SOCS-box proteins which shares 65% similarity with a related protein WSB1[13]. Northern blotting of different mouse tissues has shown that high levels of *Wsb2* transcript are present in all tissues examined, including brain, heart, and skeletal muscle [13]. It is postulated that WSB2 may act as an SR component of Cullin 5-RBX2-Elongin B/C (CRL5) E3 ubiquitin ligase complex [14], which mediates the ubiquitination and subsequent proteasomal degradation of target proteins. Only a few WSB2-targeted substrates have been identified, including the granulocyte colony-stimulating factor (G-CSF) receptor [15], interleukin-21 (IL-21) receptor [16], cyclin D1 [17], p53 [18], and lysine-methylated RelA [19]. Differential expressions of WSB2 have been reported in drug-resistant multiple myeloma cell lines [20] and human melanoma and lung cancer tissue samples [21, 22]. However, this gene has not been linked with any human disease and its role in neurodevelopment remains unexplored.

Here, we describe five patients from four unrelated families affected by developmental delays, brain anomalies, and dysmorphic features with or without intrauterine growth restriction (IUGR) and hypotonia, who were found to have homozygous, ultra-rare, predicted loss-of-function (pLoF) or missense variants in *WSB2*. We report the findings from a comprehensive phenotypic screening of the *Wsb2*-mutant (mut) mice which suggests overlapping findings between human disease and mouse model.

## Subjects and methods

### Participant identification and recruitment

This study was approved by the Institutional Review Board (IRB) at Boston Children’s Hospital (BCH at Boston, MA, USA) under the protocol 10-02-0253 and University of Miami (IRB protocol 20230140). All patients or their guardians provided written informed consent under BCH protocol 10-02-0253, collaborator protocol (HMO-0306-10), or through GeneDx protocol Research to Expand the Understanding of Genetic Variants: Clinical and Genetic Correlations, Western Institutional Review Board (protocol# 20171030). Participants were identified through the Manton Center for Orphan Disease Research, GeneDx, and GeneMatcher [23–25]. Informed consent has been obtained from all subjects or their legal guardians, and all clinical investigations adhered to the principles of the Declaration of Helsinki. All patients were examined by a clinical geneticist and/or neurologist. Pedigrees and deep phenotypic data for each patient were collected from collaborating clinicians using a standardized template. Brain magnetic resonance imaging (MRI) was collected whenever possible and reviewed by a board-certified neuroradiologist.

### Exome and genome sequencing

For the GeneDx exome sequencing cases, using genomic DNA from the patient and parents, the exonic regions and flanking splice junctions of the genome were captured using the Clinical Research Exome kit (Agilent Technologies, Santa Clara, CA) (Patient 1) or the IDT xGen Exome Research Panel v1.0 (Integrated DNA Technologies, Coralville, IA) (Patient 5). Massively parallel (NextGen) sequencing was done on an illumina system with 2x150bp paired-end reads. For exome sequencing of family 3 (Patient 3 and 4), genome capture was done using the IDT xGen Exome Research Panel v2.0 combined with xGen Human mtDNA Research Panel v1.0 (Integrated DNA Technologies), sequencing done on illumina NOVA-X. For the GeneDx genome sequencing case (Patient 2), using genomic DNA from the patient and parents, PCR-free whole genome sequencing libraries were prepared using illumina® DNA PCR-Free Library Prep following the manufacturer’s protocol (illumina, San Diego, CA). Massively parallel (NextGen) sequencing was performed on an illumina NovaSeq6000 with 2x150bp paired-end reads. Reads were aligned to human genome build GRCh37/UCSC hg19 and analyzed for sequence variants using a custom-developed analysis tool or Geneyx analysis software for secondary pipeline using DRAGEN. Reported variants were confirmed, if necessary, by an appropriate orthogonal method in the patient and, if submitted, in selected relatives. For both exome and genome cases, additional sequencing technology and variant interpretation protocol have been previously described [26]. The general assertion criteria for variant classification are publicly available on the GeneDx ClinVar submission page (http://www.ncbi.nlm.nih.gov/clinvar/submitters/26957/).

### Molecular modeling

The WSB2 protein structure model was built on the predicted 3D conformation via AlphaFold [27]. The protein structure illustration and amino acid mutations were generated by PyMOL (The PyMOL Molecular Graphics System, version 2.0 Schrödinger).

### Mouse strain and phenotyping

The *Wsb2*-mutant (mut) (C57BL/6N Charles River-*Wsb2*^tm1b(EUCOMM)Hmgu^/Ieg; EM:08073) mice were derived from the International Knockout Mouse Consortium (Knockout Mouse Project (KOMP) Repository, IKMC project 22842; https://www.mousephenotype.org/data/alleles/MGI:2144041/tm1a (EUCOMM)Hmgu), which was constructed using the IMPC ‘knockout first’ targeting strategy at Helmholtz Zentrum München, Germany.

From the age of 8–16 weeks, the *Wsb2*-mut mice were phenotyped systematically in the German Mouse Clinic (GMC) at Helmholtz Munich (Ingolstaedter Landstrasse 1, 85764, Neuherberg, Germany; www.mouseclinic.de) as described previously [28–30] and in accordance with the standardized phenotyping pipeline of the IMPC (IMPReSS: https://www.mousephenotype.org/impress/index). In brief, a cohort of 14 *Wsb2*-mut mice (7 males and 7 females in two batches) with corresponding wildtype (WT) controls (7 males and 8 females) were compared. The sample size was determined in the context of the general guidelines of the IMPC (https://www.mousephenotype.org/about-impc/animal-welfare/arrive-guidelines/) [31]. Animal numbers may vary depending on the test performed, as indicated in the text or respective figure/table legend. Body weight was measured weekly. Further phenotyping measures included growth/size/body composition, morphology, behavior, cardiovascular, craniofacial, homeostasis, muscle, reproductive, skeleton, and vision/eye. Phenotyping protocols are in agreement with the IMPC standard procedures (https://www.mousephenotype.org/impress/PipelineInfo?id=14). The method description of the tests presented in the manuscript can be found in the Supplementary Material.

Mice were housed in individually ventilated cages (IVC) available *ad libitum* according to the European Union directive 2010/63/EU, German laws, and German Mouse Clinic (GMC) housing conditions (www.mouseclinic.de). All animal care and use in this study met approval by, and complied with, the rules of the district government of Upper Bavaria (Regierung von Oberbayern) Germany and were conducted according to the rules outlined by the Helmholtz Zentrum München ethical committee.

### Statistics

Sample size was chosen according to our previous experience and common standards. If not stated otherwise, phenotypic data that were generated by the German Mouse Clinic were analyzed using automated R-scripts (version 3.2.3). Depending on parameter distribution and the questions addressed to the data, tests for genotype effects were made by using Wilcoxon rank sum test, Fisher’s exact test, analysis of variance (ANOVA) with post-hoc Tukey honestly significant difference (HSD) test and/or linear models. Where necessary, body weight was included as confounder. For categorical data, a Fisher’s exact test was used. For each normally distributed parameter, mean and standard deviation, whereas for non-normally distributed data, median, 25th percentiles and 75th percentiles were calculated. For all tests, a *p*-value < 0.05 has been used as level of significance in the comparison of phenotypic observations between mut and WT mice; a correction for multiple testing has not been performed. All data are publicly and freely available on the GMC (www.mouseclinic.de/results/phenomap-and-results/index.html) and on the IMPC portal (https://www.mousephenotype.org/data/search?term=&type=gene).

## Results

### Identification of *WSB2* variants in five patients from four families with a syndromic NDD

**Fig. 1:**
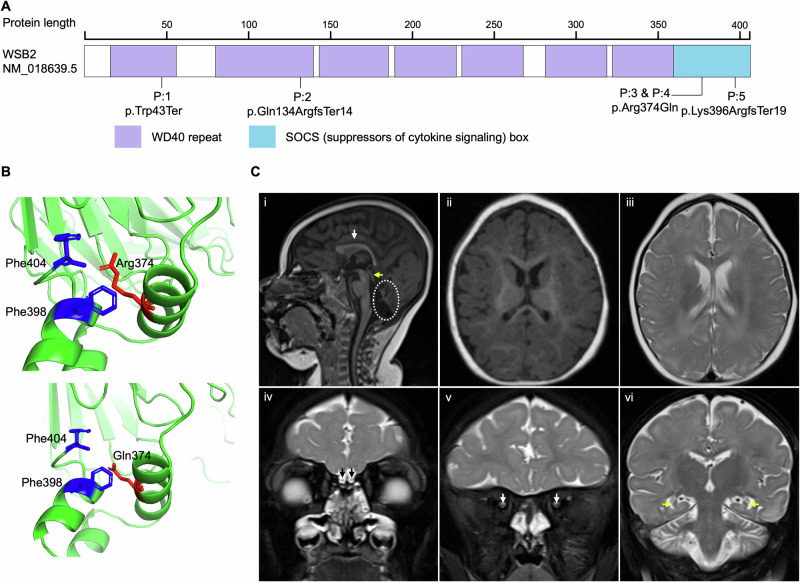
Identification of *WSB2* variants in five patients featuring syndromic NDDs. **A** Representative schematic of *WSB2* gene (NM_018639.5) containing seven WD-repeats and a suppressor of cytokine signaling (SOCS) box in the C-terminus. The *WSB2* variants are positioned in WD1, WD2, and SOCS box domain, respectively. **B** Molecular modeling of *WSB2* missense variant (NM_018639.5: c.1121G>A, p.Arg374Gln). As shown in the top panel, the wildtype residue’s (Arg374, red) positively charged side chain interacts with the aromatic rings of Phe398 and Phe404 (shown in blue), helping stabilize this region. In contrast, the side chain of Gln374 (red, bottom panel) forms weaker hydrophobic contacts with these phenylalanine, thereby potentially increasing local flexibility of the SOCS box domain. **C** Representative magnetic resonance imaging (MRI) of patient 2 with homozygous c.399del p.(Q134Rfs*14) at 12 months of age. (i) Sagittal T1-weighted MRI showing microcephaly, callosal hypogenesis (white arrow), tectal dysplasia (yellow arrow), and severe cerebellar hypoplasia/atrophy (dotted oval). (ii and iii) Axial T1- and T2-weighted MRI show undersulcation and white matter hypomyelination for age. (iv-vi) Coronal T2-weighted with fat suppression show small olfactory bulbs (black arrows), optic nerves (white arrows), and hippocampi (yellow arrows).

An international collaboration through Matchmaker Exchange [24] facilitated the identification of five patients from four unrelated families carrying homozygous predicted loss-of-function (pLoF) or missense variants in *WSB2* (Fig. 1A and Table 1), inherited from asymptomatic consanguineous parents. The variants were identified through exome or genome sequencing and confirmed by Sanger sequencing. Patient (P) 1 (P:1) harbored a nonsense variant in the exon 2 of *WSB2* (NM_018639.5: c.128G>A, p.Trp43Ter); P:2 had a single nucleotide deletion in exon 3 (NM_018639.5: c.399delG, p.Gln134ArgfsTer14), resulting in frameshift and premature stop gain; P:3 and P:4 (siblings) had the same homozygous missense variant in exon 9 (NM_018639.5: c.1121G>A, p.Arg374Gln); and P:5 had a 2-bp deletion at the end of last exon 9 (NM_018639.5: c.1187_1188delAA, p.Lys396ArgfsTer19). The variants in P:1 and P:2 were absent from the Genome Aggregation Database (gnomAD v.4.1.0), while those from P:3/P:4 and P:5 were seen only as a heterozygous variant with an extremely low frequency of 3.098 ×10^−6^ (5/1,614,188) and 1.239 ×10^−6^ (2/1,614,190), respectively. Furthermore, P:3 and P:4, siblings carrying the homozygous missense variant, had three unaffected siblings who were either heterozygous for the variant or carried the normal allele (pedigree, Supplementary Fig. S1). The *WSB2* gene is highly constrained for variants with a high probability of pLoF (pLI = 1, LOEUF = 0.363) as well as missense intolerance (missense *Z* score = 3.4). Molecular modeling was performed for the only missense variant in the cohort (p.Arg374Gln, Fig. 1B), rest being pLoF. The substitution of Gln for Arg at position 374 likely decreases the strength of the side-chain interactions between Arg374 (red) and Phe398/Phe404 (blue), which could lead to greater conformational dynamics in the SOCS box region and potentially affect WSB2’s function in E3 ubiquitin ligase complex formation.

**Table 1:** Genetic findings of patients with variants in the *WSB2* gene.

Patient #	1	2	3	4	5
Inheritance	AR	AR	AR	AR	AR
Genomic (GRCh38)	Chr12:118052364C>T	Chr12:118043160_118043161GC>G	Chr12:118034290C>T	Chr12:118034290C>T	Chr12:118034222_118034224CTT>C
cDNA(NM_018639.5)	c.128G>A	c.399delG	c.1121G>A	c.1121G>A	c.1187_1188delAA
Protein	p.Trp43Ter (p.W43X)	p.Gln134ArgfsTer14	p.Arg374Gln	p.Arg374Gln	p.Lys396ArgfsTer19
GnomAD frequency	0	0	3.098 ×10^−6^	3.098 ×10^−6^	1.239 ×10^−6^
CADD score	41	–	31	31	33
Other genetic variants	LRP2 c.10223C>T, p.P3408L, homozygous, inherited from parents, classification VUS	None reported	None reported	None reported	By clinical WES: PIEZO2 c.5167A>G, p.I1723V, heterozygous, inherited from father, VUS. By CGH microarray: 152 kb deletion of Xp22.33 (765,592–917,693). Paternally inherited. Normal with Multiple AOH

*AOH* absence of heterozygosity, *AR* autosomal recessive, *CGH* comparative genomic hybridization, *VUS* variant of uncertain significance.

The patients carrying homozygous nonsense or frameshifting variants had a more severe phenotype including global developmental delay (GDD), generalized hypotonia and muscle weakness, dysmorphic features, and microcephaly along with abnormal brain morphology on magnetic resonance imaging (MRI) (Fig. 1C; Table 2 and Supplementary Table S1). Their other clinical findings included intrauterine growth restriction (IUGR), low birth weight, and apnea. All three patients had feeding difficulties with dependency on enteral nutrition, and two patients had gastrointestinal (GI) dysmotility, expressed as dysphagia or constipation. In comparison, the two siblings (P:3 and P:4) from family 3 carrying the homozygous missense variant had a milder phenotype. They did have GDD, hypotonia, mild dysmorphic features, but no IUGR or microcephaly, and MRI performed at an early age were reported as normal (the older affected sibling’s MRI at a 11-year age was abnormal with hypoplasia of the vermis and possible heterotopia in the left frontal region). History of seizures was present in P:1, P:3 and P:5.

**Table 2:** Clinical findings of patients with variants in the *WSB2* gene.

Patient #	1	2	3	4	5
Sex	Female	Female	Male	Female	Female
Ethnicity	Middle Eastern	Amish/Mennonite	Ashkenazi Jew	Ashkenazi Jew	Emirati
Current age	4 years, 4 mo	3 years	13 years	9 years	12 years, 7 mo
GA at birth	36 weeks, 6 days	36 weeks, 6 days	39 weeks, 2 days	39 weeks, 5 days	Full term
Birth weight(with percentiles or z-score)	1.87 kg (<1%ile, z-score: −2.42)	2.09 kg (3.1%ile, z-score: −1.87)	3.0 kg (23.6%ile, z-score: −0.72)	2.926 kg (21.4%ile, z-score: −0.79)	2.3 kg (<1%ile, z-score: −2.48)
Birth length	43 cm (1.0%ile, z-score: −2.32)	41 cm (<1%ile, z-score: −3.21)	Not available	Not available	Not available
OFC birth	29 cm (<1%ile, z-score: −2.85)	29.5 cm (<1%ile, z-score: −2.50)	33 cm (18.6%ile, z-score: −0.89)	33 cm (26.7%ile, z-score: −0.62)	Not available
Neonatal complications	IUGR	IUGR	–	–	Oligohydramnios
Hypotonia	+, generalized	+, severe	+	Not available	+, severe
GDD/ID (IQ)	GDD	GDD	GDD/ID (IQ 46)	GDD/ID (IQ NA)	GDD
MRI findings	Severe microcephaly, Brain with under sulcation and gyration	Microcephaly Callosal hypogenesis Pachygyria and white matter hypomyelination Small olfactory bulbs, optic nerves, and hippocampi Tectal dysplasia Severe cerebellar hypoplasia and atrophy	Normal brain MRI at 6.5 yrs; hypoplasia of vermis at 11 yrs	Normal brain MRI at 3 yrs	Microcephaly, cerebellar hypoplasia, thin corpus callosum, and anterior horn cell disease
Seizures	+, started at age of 1.5 yrs, now free of seizure and not on medication	–	+, started at age 10 yrs and responded to treatment	–	+, started at 6 yrs, controlled with keppra and lamotrigine
Behavior abnormalities	Hand stereotypies	–	Autism	Autism, ADHD	movement disorder with near constant choreiform movements
Others	Proteinuria	–	Abnormal gait, joint hyperlaxity; EMG: motor neuron dysfunction	Joint hyperlaxity	Wenckebach second-degree heart block

*ADHD* attention-deficit/hyperactivity disorder, *EMG* electromyography, *GA* gestational age, *GDD* global developmental delay, *ID* intellectual disability, *IQ* intellectual quotient, *IUGR* intrauterine growth restriction, *MRI* magnetic resonance imaging, *OFC* occipital frontal circumference.

Various dysmorphic features included microcephaly, micrognathia, high-arched palate, foot deformity with high arch, overlapping toes, and clinodactyly of the 5th finger. Vision problems were noted in 4 out of 5 patients but they were heterogeneous, including hyperopia, ptosis, bilateral abnormal pupil morphology, and hypoplastic optic nerves. No hearing issue was reported.

### *Wsb2*-mutant mice exhibit an overlapping phenotype with human patients

**Fig. 2:**
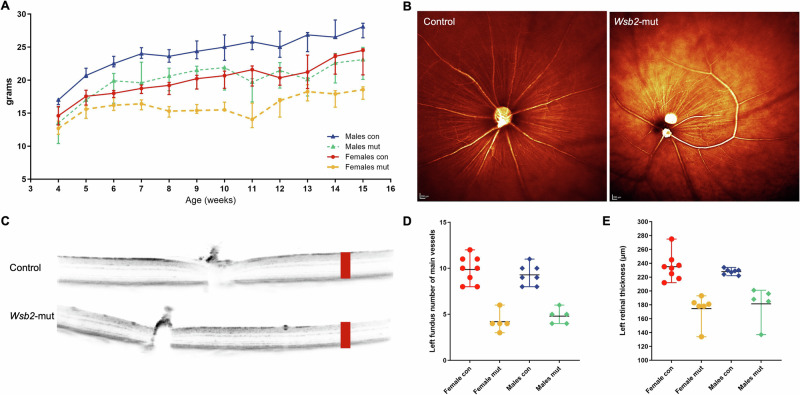
*Wsb2*-mutant mice exhibited with lower body weight and retinal abnormalities in both males and females. **A** Shows the body weight at different ages (4–15 week) between control and *Wsb2*-mut mice. *n* = 7–8 mice per group and per genotype, median and interquartile range shown. **B**–**E** Show the retinal abnormalities in *Wsb2*-mut mice, including irregular retinal blood vessel morphology (**B**, **D**) and reduced total retinal thickness (**C**, **E**) as shown in the left eye. For control group: *n* = 15, 8 females and 7 males; and for *Wsb2*-mut mice: *n* = 11, 6 females and 5 males. The right eye showed a similar phenotype. Red boxes in (**C**) represent an arbitrary evaluation of total retinal thickness, using equally sized rectangles over the retinal layers in control and *Wsb2*-mut mice to illustrate the reduction in thickness across all retinal layers.

The *Wsb2*-mutant (mut; *Wsb2*^tm1b(EUCOMM)Hmgu^, Supplementary Fig. S2) mouse line was generated and analyzed at the German Mouse Clinic [32]. Systemic phenotyping of the *Wsb2*-mut mice revealed several overlapping phenotypes with human patients affecting multiple organ systems. Compared to control (con) mice, both male (M) and female (F) mutant (mut) mice showed reduced body weight through all the phenotyping pipeline (final body weight M con vs M mut: 28.2 g vs 21.8 g; *p* = 0.001; final body weight F con vs F mut: 23.9 g vs 17.8 g; *p* < 0.001; T-test), (Fig. 2A). This finding correlated with lower food intake (M con vs M mut: 1.6 g vs 0.5 g and F con vs F mut: 2.1 g vs 0.2 g; *p* < 0.001, ANCOVA), decrease in respiratory exchange ratio (con vs mut, *p* < 0.001), and decrease in metabolic rate (con vs mut, *p* < 0.001) performed using indirect calorimetry (Supplementary Fig. S3). Dual-energy X-ray absorptiometry (DXA) analysis revealed a significantly decreased bone mineral content (BMC) and fat mass in male *Wsb2*-mut mice. Body length was slightly reduced in 14-week-old mutants, when compared to controls (M: 9.71 ± 0.30 cm vs 9.34 ± 0.45 cm; F: 9.51 ± 0.25 cm vs 9.07 ± 0.15 cm; *p* < 0.01). Upon morphological examination, 5/7 female and 4/7 male mutant mice showed abnormal upper teeth; X-ray analyses also showed a decreased size of maxillary incisors in mutant mice (3/5 female and 4/5 male mutants). Ophthalmologic examination revealed an abnormal retinal blood vessel morphology and vasculature pattern (Fig. 2B, D; *p* < 0.001) with a reduction in total retinal thickness in mutant mice compared to controls (Fig. 2C, E; mean ±SD, left eye: con 231.7 ±14.4 μm vs mut 177.6 ±22.1 μm; right eye: con 235.4 ±9.8 μm vs mut 180.5 ±22.0 μm; *p* < 0.001 Wilcoxon rank-sum test).

**Fig. 3:**
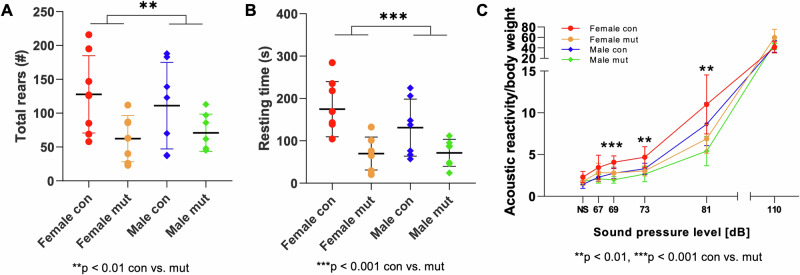
Behavioral testing revealed decreased exploratory behavior in response to a novel environment and blunted acoustic reactivity in *Wsb2*-mutant mice. In the novel open field test, the mutant mice showed decreased rearing activity (**A**) and decreased resting time (**B**) when compared to control mice. **C** Prepulse Inhibition (PPI)/Acoustic Startle Response (ASR) testing showed decreased acoustic reactivity in the mutant mice at lower non-startling sound pressure levels. Con control, mut mutant, NS no stimulus.

There were behavioral abnormalities in the *Wsb2*-mut mice such as decreased vertical exploration, as indexed by reduced rearing activity (Fig. 3A) and decreased resting time (Fig. 3B), in response to a novel mildly stressful environment (open field). Additionally, they exhibited decreased acoustic reactivity to lower sound pressure levels (Fig. 3C) in the Prepulse Inhibition (PPI)/Acoustic Startle Response (ASR) test. Further observations indicated increased locomotor activity (SHIRPA *p* = 0.03 ANOVA) and more tail elevation than control mice (92.9%, 13/14 vs 0%, 0/14, *p* < 0.00001, Fisher’s Exact test), hinting towards heightened alertness. Transthoracic echocardiography (TTE, Supplementary Fig. S4) revealed a significantly reduced heart rate in mut mice after correcting for lower body weight, confirmed by electrocardiogram. Additionally, both male and female homozygous mutant mice were found to be infertile. In the histological examination of testes, changes in testicular architecture and cell composition with severe testicular tubular atrophy and Leydig cell hyperplasia (Supplementary Fig. S5) were evident in male *Wsb2*-mut mice.

## Discussion

Cullin-RING-type ligases (CRL), the largest family of E3 ubiquitin ligases, promote the ubiquitination of approximately 20% of cellular proteins destined for degradation via ubiquitin-proteasome system [33]. Mutations in these ubiquitin ligases are associated with neurodevelopmental phenotype in humans, for instance cullin3 (CUL3) [34–36] and CUL4B [37, 38], underscoring the importance of regulated proteolysis in neurons. In this study, we present five patients with neurodevelopmental disorders (NDDs) associated with extremely rare variants in *WSB2*, encoding a CRL substrate receptor (SR) not previously linked to human disease.

The WSB2 protein has been identified as an SR of the Cullin 5-RBX2-Elongin B/C (CRL5) E3 ubiquitin ligase complex [14] characterized by the presence of two conserved domains, the N-terminal 7 WD-40 repeat and the C-terminal suppressor of cytokine signaling (SOCS)-box domain. A (SOCS)-box consists of a BC box for Elongin B/C binding [39] and a CUL5 box for CUL5 binding via its amino acid sequence LPΦP (Φ represents a hydrophobic residue) [14], whereas the WD-40 domain serves as a rigid scaffold for protein-protein interactions [40]. It is suspected that the stop-gain (in P:1) and frameshift (in P:2) variants will cause nonsense-mediated mRNA decay (NMD) and loss of function of WSB2, whereas the missense (in P:3 and P:4) and frameshift variant (in P:5) in SOCS-box domain may disrupt its protein interaction with the CRL5 complex, thus affecting their ability to ubiquitinate and degrade its protein targets.

All patients in this study exhibited global developmental delay and four of them showed abnormal brain morphology on MRI. Single-cell RNA sequencing data from human samples indicate that the major isoform of human *WSB2* gene (NM_018639.5) is ubiquitously expressed across various brain regions, with a notable enrichment in the neocortex after late fetal period (Supplementary Fig. S6). Consistently, *Wsb2*-mutant mice exhibited behavioral abnormalities, including hyperactivity, altered exploration, and hyper alertness, although potential structural brain changes remain unknown. Similarly, the low birth weight and failure to thrive phenotype seen in three of the five patients (P:1, P:2 and P:5) carrying homozygous pLoF variants was also seen in both male and female *Wsb2*-mutant mice. The visual findings in human patients are relatively mild and heterogeneous (Supplementary Table S1) but the retinal abnormalities seen in *Wsb2*-mutant mutant, characterized by decreased retinal thickness and abnormal retinal blood vessel morphology and vasculature patterns suggests the need for future ophthalmology evaluation. Similarly, future cardiac evaluation should be considered as patient P:5 has been diagnosed with Wenckebach second-degree heart block at 8 year of age and lower heart rates have been noted in *Wsb2*-mut mice.

WSB2 may regulate various developmental and physiological processes given its role in ubiquitination and its widespread expression. The phenotypic findings from human patients and mutant mice suggest that it may be involved in growth regulation, neurodevelopment, retinal vascular development, and autonomic regulation, though the precise molecular mechanisms remain to be elucidated. Previous studies on WSB2 have primarily concentrated on its expression in cancer tissue and cells, as well as its roles in carcinogenesis [20–22]. From these studies, a limited number of WSB2 substrates have been characterized, including the G-CSF receptor and IL-21 receptor in immune cells [15, 16], cyclin D1 (Ccnd1) [17], p53 [18], and chromatin-bound methylated RelA [19], summarized in Supplementary Table S2. Among these substrates, Ccnd1, along with its catalytic counterpart cyclin-dependent kinase 4 (Cdk4), plays a crucial role in regulating G_1_ phase length and influences the decision of neural stem cells to proliferate or differentiate [41–44]. Overexpression of Ccnd1-Cdk4 shortens G1 phase, delays neurogenesis, and promotes the generation and expansion of basal progenitors [43]. Similarly, the transcription factor p53, a well-known tumor suppressor [45, 46], is broadly expressed in the brain and its excessive activation can lead to developmental defects and increased neuronal cell death in various human diseases, correlating with a range of phenotypes, including craniofacial, cardiovascular, and neuronal defects [47–55]. Beyond these substrates, other potentially critical proteins that may be regulated by WSB2 are yet to be determined.

In summary, our study provides evidence that recessive *WSB2* variants result in a new neurodevelopmental disorder characterized by global developmental delays, hypotonia, seizures, abnormal brain morphology, dysmorphic features, and failure to thrive. Future in vitro studies and mouse models of human *WSB2* mutations are needed to further elucidate WSB2 function, its downstream targets, and the precise mechanisms by which *WSB2* mutations contribute to disease pathogenesis.

## Supplementary information

Supplementary materials

## Data Availability

Raw data were generated at GeneDx, LLC and the German Mouse Clinic. Derived data supporting the findings of this study are available from the corresponding author [PBA and MHdA] on request. The mouse phenotyping data are available at the International Mouse Phenotyping Consortium platform (IMPC) (https://www.mousephenotype.org/data/genes/MGI:2144041).
